# Positive Regulation of Spoilage Potential and Biofilm Formation in *Shewanella baltica* OS155 via Quorum Sensing System Composed of DKP and Orphan LuxRs

**DOI:** 10.3389/fmicb.2019.00135

**Published:** 2019-02-05

**Authors:** Yanbo Wang, Feifei Wang, Chong Wang, Xiuting Li, Linglin Fu

**Affiliations:** ^1^Food Safety Key Laboratory of Zhejiang Province, School of Food Science and Biotechnology, Zhejiang Gongshang University, Hangzhou, China; ^2^Zhejiang Engineering Institute of Food Quality and Safety, Zhejiang Gongshang University, Hangzhou, China; ^3^Beijing Advanced Innovation Center for Food Nutrition and Human Health, Beijing Technology and Business University, Beijing, China

**Keywords:** quorum sensing, diketopiperazines, orphan LuxR-type proteins, spoilage, biofilm formation

## Abstract

The spoilage potential and biofilm formation of *Shewanella baltica* are reported to be regulated by Quorum sensing (QS) system from the phenotype point of view, but the specific mechanism is not fully understood. In the present study, the QS autoinducers were detected by UHPLC-MS/MS, cell density-dependent *luxR*-type genes were obtained through autoregulation experiments among a series of candidates in *S. baltica* OS155 (The SSO of large yellow croaker). The direct interaction between cyclo-(L-Pro-L-Phe) (PP) and LuxR01 as well as LuxR02 proteins was revealed via *in vitro* binding assay. Deletion of *luxR*-type genes (*luxR01* and *luxR02*) impaired spoilage potential and biofilm formation of *S. baltica* OS155 in various degrees. Transcriptional analysis and qRT-PCR validation showed that spoilage and biofilm-related genes *torS, speF*, and *pomA* were down-regulated in *luxR*01 and *luxR*02 deletion strains. In addition, exogenous PP promoted spoilage potential and biofilm formation, which could be attenuated by *luxR*01 or *luxR*02 deletion. Our results revealed an explicit QS system employing PP as autoinducer and two orphan LuxRs as receptors which positively regulated spoilage capacity and biofilm formation via transcriptional regulation of corresponding genes in *S. baltica* OS155, which provides potential specific targets for seafood preservation involving QS system.

## Introduction

Seafood is one of the most highly perishable food products because of the chemical effects of atmospheric oxygen and the growth of spoilage microorganisms (Özogul et al., 2004). Bacterial metabolism caused by microbial growth is the major cause of seafood spoilage and results in the unacceptable characteristics including quality and safety impairment (Robson et al., 2007). However, only a portion of the original seafood microbiota, named SSOs, has strong adaptability to storage conditions, and thus prevail over the rest of the microbiota, reaching high populations and producing corresponding spoilage products (Parlapani et al., 2015). Biochemical analysis on the basis of nucleotide metabolism, production of TMA, hypoxanthine (Hx), total volatile basic nitrogen (TVB-N), and BAs have been commonly utilized to estimate fish quality. Large yellow croaker (*Pseudosciaena crocea*) is commercially important because of its delicious taste and good nutrition, representing one of the largest yield of single species in marine net-cage farming in China. *Shewanella baltica*, a commonly dominant spoilage bacteria in seafood products such as iced sea salmon (Hozbor et al., 2006), gutted sea bass (Parlapani et al., 2015), chilled fresh Mediterranean swordfish (Pantazi et al., 2008) and tropical prawns (Chinivasagam et al., 1998; Zhu et al., 2015), has received increasing attention in recent years due to its significant role in seafood spoilage, and has also been identified as the SSO of large yellow croaker stored at 4°C (Gu et al., 2013; Zhu et al., 2016).

The term QS was first put forward by Fuqua et al. (1994). QS is a cell density-dependent signaling system in various bacterial species (Fuqua et al., 1994; Bassler and Losick, 2006). These bacteria autonomously sense a threshold concentration of the autoinducers (corresponding to a critical cell density) produced by the microbial community, leading to gene expression alterations through transcriptional regulators, and thus regulate their behaviors to be beneficial to survival (Kievit and Iglewski, 2000; Smith et al., 2004). Therefore, QS-controlled physiological activities, such as bioluminescence, virulence factors secretion, biofilm formation, sporulation and so on, require the collective action of the group to be effective (Bassler and Losick, 2006; Papenfort and Bassler, 2016). Gram-positive bacteria employ oligopeptide as QS autoinducers and membrane-bound sensor histidine kinases as QS receptors, and Gram-negative bacteria generally utilize LuxIR-type proteins sensing *N*-acyl-homoserine lactones (AHLs) signals primarily for intraspecies communication (Whitehead et al., 2001; Waters and Bassler, 2005). Moreover, LuxS/Autoinducer-2 (AI-2) has been supposed to be universal signal in both Gram-negative and Gram-positive bacteria (Smith et al., 2004), which has been observed in several bacteria (Schauder and Bassler, 2001; Taga et al., 2001; Duan et al., 2003). Recently, a range of cyclic dipeptides (diketopiperazines, DKPs) produced by multiple Gram-negative bacteria have been reported to modulate AHLs-specific sensor system (Holden et al., 1999; Degrassi et al., 2002; Park et al., 2006), previous studies have demonstrated the positive role of DKPs as QS signals in promoting biofilm formation (Zhu et al., 2015, 2016), *torA* and ODC genes expression (Zhu et al., 2016) as well as the production of TVB-N, TMA, putrescine and extracellular proteases (Gu et al., 2013; Zhu et al., 2015, 2016). As a result, DKPs have been proposed to represent a novel naturally occurring QS autoinducers which can act as intra- and inter-species regulators (Shiner et al., 2005; Klose, 2006). In addition, 2698 of the 3550 *luxR* genes in the NCBI databases have been found as orphans which had no adjacent *luxI* gene in the chromosome according to a survey conducted by Hudaiberdiev et al. (2015), many of which were devoid of the AHL-binding motifs, thus may responded to exogenous signals besides AHL type molecules (Hudaiberdiev et al., 2015). These results provide strong evidence for the alleged role of DKPs in bacterial QS system, however, Campbell et al. (2009) found that the native DKPs exhibited neither antagonistic nor agonistic activities in reporter strains, and suggested that non-native DKPs influenced QS-regulated outcomes not through the direct interaction with LuxR-type proteins (Campbell et al., 2009). Therefore, questions remain on the genuine physiological function of DKPs in bacteria.

The QS systems involved in the physiological and clinical aspects of bacteria have attracted considerable attention and have been widely investigated (Ruffin et al., 2016; Wu et al., 2016). However, there is a lack of knowledge on the role of QS in food spoilage, especially in seafood. As cell–cell communication commonly exists in bacteria, QS should participate in the microbial ecology of food, and studies on the spoilage aspect of QS have been carried out in the past few years. It has been reported that bacterial spoilage of bean sprouts was affected by QS (Rasch et al., 2005), and milk spoilage by psychrotrophic bacteria *Pseudomonas* spp., *Serratia* spp., *Enterobacter* spp., and *Hafnia alvei* was also resulted from QS (Pinto et al., 2007). AHL-modulated exoenzyme activities have been reported in *Serratia proteamaculans* B5a isolated from cold-smoked salmon (Christensen et al., 2003), similarly, C4-HSL increased exoenzyme production in *Pseudomonas psychrophila* PSPF19 and promoted the spoilage of refrigerated freshwater fish (Bai and Rai Vittal, 2014). However, the molecular mechanism of how QS system regulates seafood spoilage remains to be revealed.

Bacterial biofilms are assemblages of bacterial cells with self-produced polymeric matrix or glycocalyx adherent to surface or interface (Costerton et al., 1999; Hall-Stoodley et al., 2004). QS has been reported to regulate the biofilm formation in pathogens, such as *Pseudomonas aeruginosa* (Davies et al., 1998), *Streptococcus mutans* (Li et al., 2002), *Staphylococcus aureus* (Yarwood et al., 2004) and *Streptococcus pneumoniae* (Vidal et al., 2011). Recently, AHLs and DKPs have been reported to promote the formation of biofilms in spoilage bacteria *Pseudomonas psychrophila* PSPF19 and *S. baltica* (Bai and Rai Vittal, 2014; Zhu et al., 2015, 2016). However, it remains unknown how biofilm is regulated by QS system in spoilage microorganisms.

*Shewanella baltica* is a facultative anaerobic, rods-shaped, and psychrotrophic Gram-negative bacterium with the optimal growth temperature around 25°C but can also grow at 0°C (Francoise, 2010). As the SSO of refrigerated shrimp and large yellow croaker, *S. baltica* produced a series of DKPs which could promote their spoilage capacity (Gu et al., 2013; Zhu et al., 2015, 2016), but the interaction between DKPs and typical QS receptors LuxR-type proteins (Holden et al., 1999; Degrassi et al., 2002; Park et al., 2006) has yet to be studied. In this study, we identified a series of orphan *luxR*-type genes by homology search within the representative strain *S. baltica* OS678 in NCBI database. Then we demonstrated the direct interaction between PP and LuxR-type protein (LuxR01 and LuxR02) in *S. baltica*. After constructing a series of orphan *luxR*-type gene deletion mutants (SB7301, SB7302, SB7303, SB7304, SB7305, SB7306, and SB7307), we demonstrated that the spoilage potential and biofilm formation were positively regulated by DKP-LuxR type QS system via governing the expression of *torS, speF*, and *pomA genes*. This work improved our understanding of the QS system employed in *S. baltica* and provided potential targets for controlling undesirable bacteria growth in foods.

## Materials and Methods

### Bacterial Strains, Plasmids and Culture Conditions

*Shewanella baltica* 73 strain was demonstrated as the SSO of large yellow croaker (*Pseudosciaene crocea*) (Gu et al., 2013), which showed 99% identity with *S. baltica* OS155 (NCBI Taxonomy ID: 325240). Thus, the target strain in this study was uniformly named as *S. baltica* OS155. All bacterial strains and plasmids involved in this study are listed in Supplementary Table S1.

*Escherichia coli* and *S. baltica* strains were grown in Luria-Bertani (LB) medium at 37 and 30°C, respectively. When necessary, additives are used as follows: DAP, 50 μg/mL; Gm, 15 μg/mL; ampicillin, 100 μg/mL; IPTG, 0.1 mM.

### Identification of Provisional *luxR*-Type Genes, Spoilage- and Biofilm-Related Genes in *S. baltica* OS155

On the basis that *S. baltica* OS678 is the representative strain of *S. baltica* species in NCBI database, we identified target genes in *S. baltica* OS155 by homology search with *S. baltica* OS678. The identification of these genes was listed in Supplementary Table S2. Among them, all of the chosen *luxR*-type genes contain sequence encoding a DNA-binding helix-turn-helix domain characteristic of genuine *luxR* genes and the conserved domains in each LuxR-type proteins were shown in Supplementary Figure S1.

### AHLs and DKPs Detection

The AHL standards (95% or higher purity), including C4-HSL, C6-HSL, C8-HSL, C10-HSL, C12-HSL, C14-HSL, 3-oxo-C8-HSL, 3-oxo-C10-HSL, 3-oxo-C12-HSL, and 3-oxo-C14-HSL were obtained from Sigma-Aldrich (St. Louis, MO, United States). The three cyclic dipeptides (DKPs) including PL, LL and PP were synthesized by Sangon Biotech Company (Shanghai, China). Reference standards were prepared as 1 μg/mL solution in methanol. *S. baltica* strains were grown in LB medium overnight (postexponential phase) at 30°C. Culture supernatants were extracted three times with equal volumes of acidified ethyl acetate (0.5% formic acid), and the extracts were evaporated under nitrogen flow (Rasch et al., 2005). Fish organs were sampled after storage at room temperature for 48 h and extracted as previously described (Buch et al., 2003). The evaporated extracts were redissolved in 1 mL methanol and stored at -20°C for further analysis using UHPLC-MS/MS. Mass spectra were recorded by means of a triple-quadrupole tandem mass spectrometer (TSQ Quantiva, Thermo Fisher Scientific, Waltham, MA, United States) coupled to a Surveyor high performance liquid chromatography system (Thermo Fisher Scientific, Waltham, MA, United States) which was equipped with a thermostated (4°C) autosampler and a 150 mm × 2.0 mm i.d., 1.8 μm Synergi Hydro RPHPLC column (Phenomenex, Aschaffenburg, Germany) kept at 30°C. The mobile phase was composed of formic acid in water (0.1%; A) and methanol (100%; B). The LC gradient was listed in Supplementary Table S3. The mass spectrometer was operated in the positive electrospray ionization mode (H-ESI) with a spray voltage of 3500 V. The temperature of the capillary was 350°C, and the sheath and auxiliary gas (nitrogen) were adjusted to 45 and 12 arbitrary units, respectively. The retention times and MS parameters used for identification and quantification of AHLs and DKPs were listed in Table 1.

**Table 1:** MS conditions used for AHLs and DKPs analysis, and the identification and quantification of AHLs and DKPs in *Shewanella baltica* OS155 and spoiled large yellow croaker.

Compound	Precursor ion (m/z)	Product ion (m/z)	Retention time (min)	Content in *Shewanella baltica* OS155 (ng/mL)	Content in spoiled large yellow croaker (ng/g)
C4-HSL	172	71, 102	1.94	/	40.20
C6-HSL	200	99, 102	5.56	/	0.63
C8-HSL	228	102, 127	6.94	/	/
C10-HSL	256	102, 155	7.66	/	/
C12-HSL	284	102, 183	8.18	/	/
C14-HSL	312	102, 211	8.71	/	/
3-oxo-C8-HSL	242	102, 141	5.92	/	/
3-oxo-C10-HSL	270	102, 169	7.07	/	/
3-oxo-C12-HSL	298	102, 197	7.73	/	/
3-oxo-C14-HSL	326	102, 225	8.24	/	/
Cyclo-(L-Pro-L-Leu)	211	70, 183	5.19	4528.10	230.57
Cyclo-(L-Pro-L-Leu)	227	86, 154	6.59	257.00	5.80
Cyclo-(L-Pro-L-Phe)	245	70, 120	5.60	3657.70	37.63

### RNA Extraction and qRT-PCR

RNA was extracted from postexponential cultures using an RNeasy mini kit (Qiagen) according to the manufacturer’s instructions. Genomic DNA contamination was eliminated by treating total RNA with Turbo DNA free (Thermo Fisher Scientific, Waltham, MA, United States). cDNA was synthesized using random hexamers (GE Healthcare) and SuperScript II reverse transcriptase (Invitrogen, Grand Island, NY, United States). qRT-PCR was performed as described previously (Burr et al., 2006). The relative gene expression was calculated using the critical threshold (ΔΔ*C*t) method with 16S rRNA being the internal control.

### Self-Induced Experiment in *S. baltica* OS155

The late exponential culture supernatant of *S. baltica* OS155 was harvested by centrifugation and subject to a 0.22 μm filter to remove all remaining cells, then filtered supernatants were dried and resuspended in PBS buffer to a tenth of the original volume (10×). The early exponential cells of *S. baltica* OS155 were treated with late exponential culture extract of a final 1× concentration (Balaban and Novick, 1995). Equal volume of PBS was used as a control. The cells were further cultured for 3 h, then the expression level of provisional *luxR*-type genes (*luxR01, luxR02, luxR03, luxR04, luxR05*, and *luxR06* genes) were quantified by qRT-PCR.

### Expression and Purification of Provisional LuxR-Type Proteins

The provisional LuxR-type proteins-coding sequences (*luxR01, luxR02, luxR03, luxR04, luxR05*, and *luxR06* genes) were fused with N-terminal FLAG tag (GACTACAAGGACGACGATGACAAG) and cloned into the expression vector pET-15b at the site of *Nde* I and *Bam*H I using One Step Cloning Kit (Vazyme, Hangzhou, China). Target genes and primers used are listed in Supplementary Table S4. The recombinant vectors were verified by sequencing and transformed into the host *E. coli* BL21 (DE3) for protein expression. The empty vector pET-15b was served as a control. The recombinant strains were cultured in LB medium with ampicillin at 37°C for 4 h. The protein expression was induced with 0.1 mM IPTG overnight at 16°C (Kleber-Janke and Becker, 2000). Cells were collected and treated with B-PER Complete Bacterial Protein Extraction Reagent (Thermo Fisher Scientific, Waltham, MA, United States) for mild protein extraction according to the manufacturer’s instruction. The recombinant proteins were purified using ANTI-FLAG M2 magnetic beads (Sigma-Aldrich, St. Louis, MO, United States) based on the specific binding between M2 antibody and FLAG tag (Brizzard et al., 1994). The purified target proteins and the vector control were analyzed by SDS-PAGE and Coomassie brilliant blue R-250 staining (Bio-Rad, Hercules, CA, United States).

### Binding Assay of LuxR-Type Proteins Versus Hypothetical Autoinducers

The purified beads-FLAG-LuxRs complexes (LuxR01, LuxR02, LuxR03, LuxR04, LuxR05, and LuxR06 proteins) were treated with a mixture containing 1 μM C4-HSL, C6-HSL, PL, LL, and PP in PBS buffer, respectively. The binding system was incubated at 4°C for 1 h with gentle rotating. The binding complexes were collected and washed with PBS buffer to remove all of the non-specifically bound molecules. The target complexes were resuspended in PBS buffer and boiled three times to release the bound molecules into the supernatant, which was collected and extracted twice with ethyl acetate. The ethyl acetate fractions were pooled, evaporated under nitrogen flow to dryness, and finally resuspended in 1 mL methanol. The empty vector pET-15b was served as a control.

### Construction of *luxR*-Type Gene Deletion Mutants

In-frame deletion mutants (*luxR01, luxR02, luxR03, luxR04, luxR05*, and *luxR06* genes deletion mutants) were constructed as reported previously (Gao et al., 2006; Jin et al., 2013). In brief, *attB1* and *attB2* sequences were flanking the specific sequences fused by the upstream and downstream regions of the target genes (in-frame deletion construct) for subsequent *attB*-*attP* (BP) recombination with pHGM01. Target genes and primers used are listed in Supplementary Table S4. The resulting recombination mixture was transformed into the donor *E. coli* WM3064, the fragment between *attP1* and *attP2* site within pHGM01 was replaced by the in-frame deletion construct, removing the *ccdB* suicide gene, thus *E. coli* WM3064 was allowed to grow on plates containing Gm and DAP. Integration of the recombinant vectors into the *S. baltica* OS155 chromosome from *E. coli* WM3064 was carried out by conjugation at a ratio of 2:1 (donor:recipient). PCR-verified transconjugants were grown in NaCl-less LB liquid overnight and then plated onto NaCl-less LB agar containing 10% sucrose. At last, Gm-sensitive and sucrose-resistant colonies were screened by PCR as deletion mutants, which were further verified through DNA sequencing. The constructed in-frame deletion strains for *luxR01, luxR02, luxR03, luxR04, luxR05*, and *luxR06* genes were named SB7301, SB7302, SB7303, SB7304, SB7305, and SB7306 mutants, respectively. The constructed in-frame deletion strain for both *luxR01* and *luxR02* genes was named SB7307. Deletion mutants were screened by PCR amplification using the outside primers (5′-O and 3′-O) and verified by DNA sequencing (Supplementary Figure S2).

### TMA Analysis

For the TMA analysis, *S. baltica* OS155 wild-type strain and all gene deletion mutants (SB7301, SB7302, SB7303, SB7304, SB7305, SB7306, and SB7307) were cultured in the LB broth with 10 and 100 mM TMAO, respectively. The TMA was quantified spectrophotometrically by following the colorimetric formation of the picric acid salt of TMA (Dyer, 1945). The wild-type strain was used as control and water was used as blank.

### Putrescine Analysis

For putrescine analysis, *S. baltica* OS155 wild-type strain and all gene deletion mutants (SB7301, SB7302, SB7303, SB7304, SB7305, SB7306, and SB7307) were cultured in LB broth with 0.5/2% L-Ornithine monohydrochloride and 0.5/2% L-Arginine monohydrochloride supplemented with 0.005% pyridoxal-5′-phosphate. The postexponential cell culture supernatant was obtained as test samples by centrifuging at 5,000 *g* for 10 min at 4°C. Putrescine standard was purchased from Sigma-Aldrich (St. Louis, MO, United States), the standard solutions were prepared in 0.1 M HCL to a final concentration of 0.0, 0.1, 0.5, 1.0, 5.0, 12.5, 25, and 50 μg/ml, respectively. The extraction and pre-column derivatization of test samples or BA standards were carried out based on the methods developed by Kim et al. (2009). BAs were measured using HPLC according to the procedures reported by Ben-Gigirey et al. (1998) with little modification. The wild-type strain was used as control.

### Static Biofilm Formation Analysis

The static biofilm formation in *S. baltica* OS155 wild-type strain and all gene deletion mutants (SB7301, SB7302, SB7303, SB7304, SB7305, and SB7306) was quantified by microtiter plate assay as described previously with some modifications (Djordjevic et al., 2002). Overnight cultures of target strains were diluted at 1:100 ratio in a fresh sterile LB medium and then transferred into 96-well microtiter plates. After further incubation without shaking at 30°C for 60 h, LB medium was removed and the plates were washed thrice with sterile distilled water. The plates were air-dried and stained with 1% crystal violet solution in water for 20 min. The wells were washed thoroughly with sterile distilled water, after drying, 95% ethanol was used to dissolve the stained biofilm. The absorbance was measured at 595 nm using a microplate reader. Wild-type strain was used as control and pure LB liquid medium was used as blank.

The density and thickness of biofilm were analyzed by CLSM. Overnight cultures of target strains were diluted at 1:100 ratio in a fresh sterile LB medium. 2 mL dilutions were transferred into the 35 mm glass bottom culture dishes with 20 mm micro-well (Cellvis, Sunnyvale, CA, United States). After further incubation without shaking at 30°C for 60 h, the non-adhering cells were removed by washing thrice with sterile distilled water. Biofilms were fluorescent-labeled with Syto 9 dye, a green cell-permeant nucleic acid marker (Syto 9) in LIVE/DEAD *Bac*Light Bacterial Viability kit (Invitrogen, Grand Island, NY, United States), following the manufacturer’s instructions for biofilm staining. Image acquisition was performed using a Zeiss LSM 800 Confocal Laser Scanning Microscope (Zeiss, Jena, Germany). All biofilms were scanned at 800 Hz using a 63× oil immersion objective lens with a 488-nm argon laser set at 25% intensity. A range of 500–600 nm was chosen to collect the Syto 9 emission fluorescence. Two stacks of horizontal plane images with a z-step of 1 μm were acquired per dish. Three-dimensional projections of the biofilms and the maximum thickness were acquired for three biofilm replicas per strain using IMARIS 7.1 software (Bitplane, Switzerland) (Guilbaud et al., 2015).

### The Effects of Exogenous PP on TMA, Putrescine and Biofilm Formation in *S. baltica* OS155

Exogenous PP was added at 10 μM when cells were grown to early exponential phase (about 3 h), an equal volume of DMSO was used as control. After further culture for 3 h, TMA, putrescine and biofilm formation analyses were carried out as described above.

### Transcriptome Profiling of Wild-Type Strain and SB7301 Mutant

Wild-type strain and SB7301 mutant (which is of the same status as SB7302 according to our previous results) were grown overnight and total RNA was extracted from the cell pellets. RNA samples were treated with Ribo-Zero Magnetic kit to remove ribosomal RNA. RNA transcripts were fragmented using Truseq™ RNA sample prep Kit for Library construction. The fragmented RNA sequences were reverse transcribed, random primers were used to synthesize the second cDNA strand. After adapter ligation, the second cDNA strand was digested with uracil-DNA glycosylase. The remaining fragments were PCR amplified for 15 cycles and the amplicons were selected by 2% agarose gel electrophoresis. The cDNA products were subjected to high-throughput sequencing with an Illumina HiSeq 2000 sequencer to obtain single reads of 100 bp using the forward sequencing primer.

### Statistical Analyses

All the statistical results were analyzed with three replicates according to a completely randomized design. The Student’s *t*-test was employed to find out significance difference between the means. Data were analyzed statistically by repeated measures using SPSS 17.0 procedures and *p* < 0.05 was considered as statistically significant.

## Results

### Identification and Quantification of Autoinducer Candidates

Our previous study has isolated *S. baltica* OS155 from large yellow croaker and identified it as the SSO during low-temperature storage. To better investigate the QS system in *S. baltica* OS155, we first identified and quantified the hypothetical autoinducers produced by *S. baltica* OS155. Using the previously described UHPLC-MS/MS method (Wang et al., 2017), all AHLs and DKPs standards were separated within 9 min. Specifically, PL, LL, and PP were detected in the overnight culture supernatant of *S. baltica* OS155 in LB medium (Supplementary Figure S3A), and C4-HSL, C6-HSL, PL, LL, and PP were detected in spoiled large yellow croaker (Supplementary Figure S3B). In most situations, DKPs showed a much higher concentration than AHLs and thus were assumed to be the predominant autoinducers in both *S. baltica* OS155 and spoiled large yellow croaker (Table 1).

### Identification of Cell Density-Dependent LuxR-Type Proteins in *S. baltica* OS155

**FIGURE 1:**
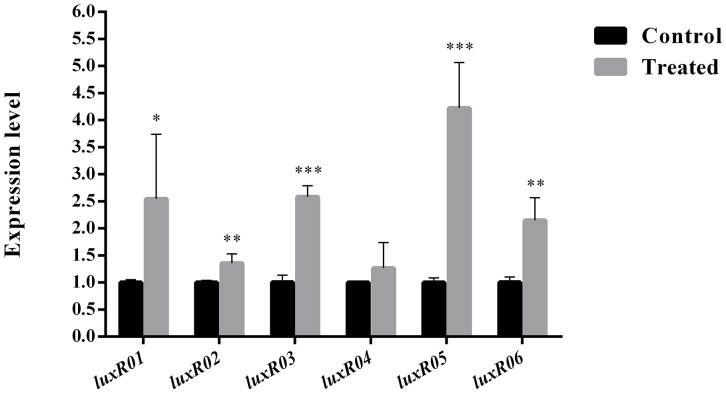
Identification of cell density-dependent LuxR-type proteins in *Shewanella baltica* OS155. Effects of post exponential supernatant extract on the transcription of provisional *luxR*-type genes, including *luxR01, luxR02, luxR03, luxR04, luxR05* and *luxR06*, were quantified by qRT-PCR in *S. baltica* OS155. Data was presented as the mean ± standard deviation (*n* = 3, ^∗^*p* < 0.05, ^∗∗^*p* < 0.01, ^∗∗∗^*p* < 0.001).

Most QS autoinducers are accumulated at high cell density, and the activated LuxR-type proteins act as transcription regulators to control the expression of relevant genes including themselves in a feedback loop. Based on this, we first introduced postexponential (high cell density) supernatants of *S. baltica* OS155 into their early exponential phase (low cell density) culture and analyzed the subsequent gene expression alteration. By qRT-PCR and statistical analyses, we found that *luxR01, luxR02, luxR03, luxR05*, and *luxR06* genes were up-regulated in different degrees with statistical significance when induced by self-culture supernatant (Figure 1). This result preliminarily demonstrated *luxR01, luxR02, luxR03, luxR05*, and *luxR06* genes as genuine *luxR*-type genes in QS system of *S. baltica* OS155.

### The Interaction Between LuxR-Type Proteins and Hypothetical Autoinducers

**FIGURE 2:**
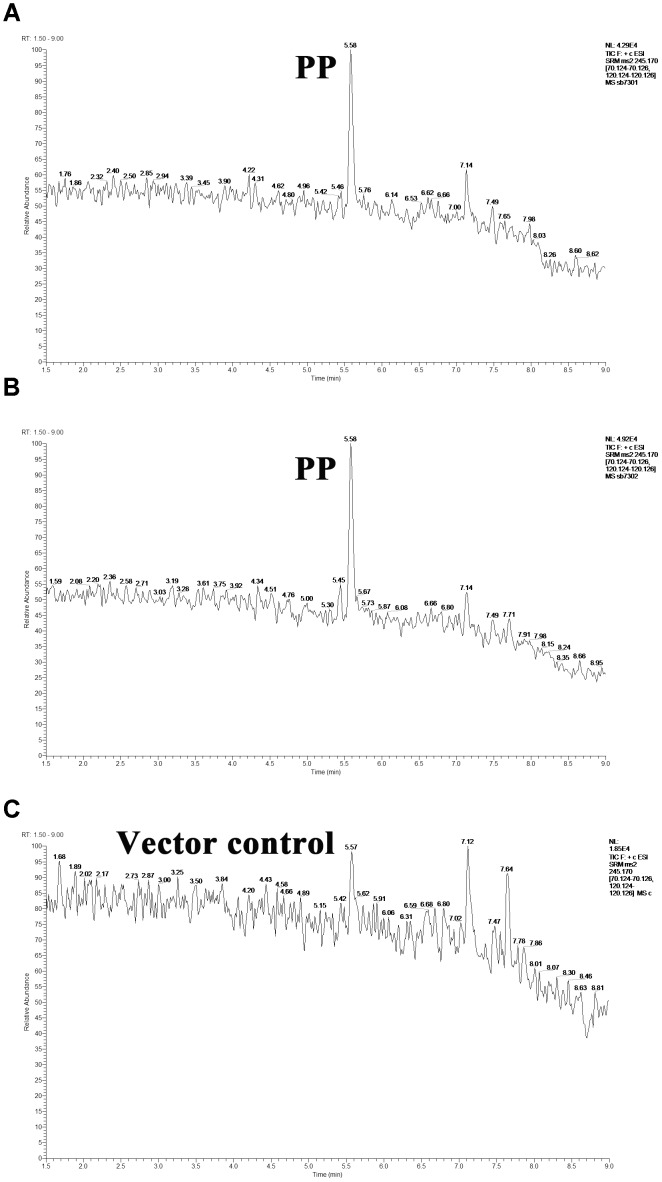
The direct interaction between cyclo-(L-Pro-L-Phe) (PP) molecule and LuxR01 as well as LuxR02 proteins. PP molecule captured by purified LuxR01 **(A)** and LuxR02 **(B)** in the mixture of autoinducer candidates was detected using UHPLC-MS/MS as compared with the pET-15b vector control **(C)**. PP: cyclo-(L-Pro-L-Phe).

LuxR-type protein is typical QS receptor in Gram-negative bacteria whose activity is modulated by autoinducer. In light of this, we evaluated the interaction between the detected hypothetical autoinducers and LuxR-type proteins in *S. baltica* OS155. The purified six LuxR-type proteins were verified by SDS-PAGE and Coomassie brilliant blue staining (Supplementary Figure S4). We introduced the target proteins-bound magnetic beads into the intermixture of hypothetical QS autoinducers to enrich the specific autoinducers, which were further extracted by ethyl acetate and subject to UHPLC-MS/MS for identification. As shown in Figure 2, PP was detected in LuxR01 and LuxR02 protein binding system as compared with the pET-15b vector control, yet other LuxR-type proteins were failed to bind any hypothetical autoinducers. Our data suggested that PP is an autoinducer recognized by LuxR01 and LuxR02 proteins in *S. baltica* OS155.

### Spoilage Potential of Deletion Strains

**FIGURE 3:**
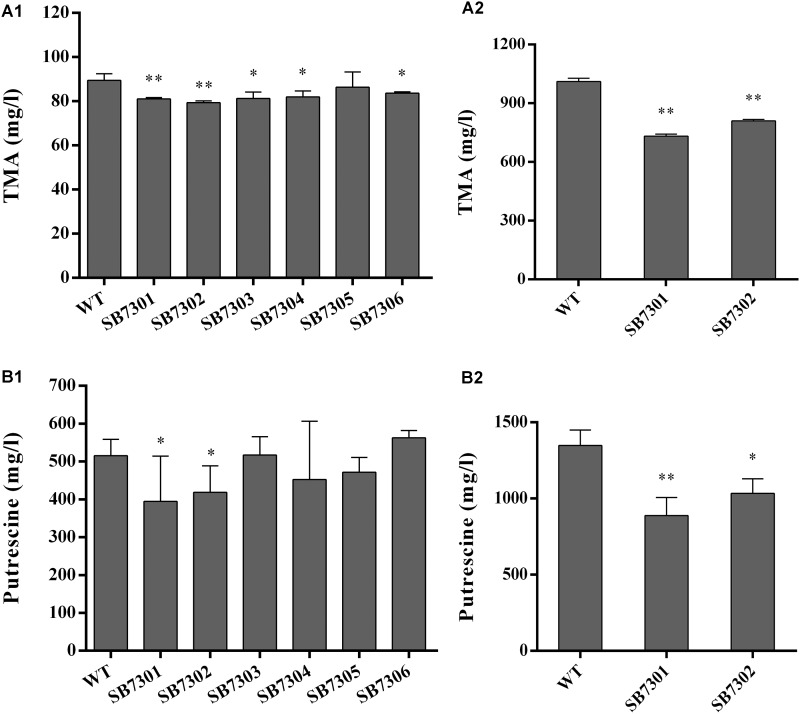
Spoilage potential of gene deletion mutants. The TMA and putrescine production of wild-type and each deletion mutant (SB7301, SB7302, SB7303, SB7304, SB7305, and SB7306) strain were measured at the addition of 10 mM **(A1)** and 100 mM **(A2)** TMAO, 0.5% **(B1)**, and 2% **(B2)** L-Ornithine monohydrochloride and L-Arginine monohydrochloride, respectively. Data was presented as the mean ± standard deviation (*n* = 3, ^∗^*p* < 0.05, ^∗∗^*p* < 0.01).

It has been reported that QS system underpins the spoilage potential of spoilage microorganisms. On this basis, we explored the role of each LuxR-type protein in regulating spoilage-related activity, including TMA and putrescine production. We established SB7301, SB7302, SB7303, SB7304, SB7305, SB7306, and SB7307 mutans, and tested the spoilage potential of these strains. As shown in Figure 3A1,A2, all mutant strains except for SB7305 were partially decreased in TMA production compared with wide type strain. However, putrescine production was reduced only in SB7301 and SB7302 mutants (Figure 3B1,B2). In addition, the reduction of TMA and putrescine production in SB7301 and SB7302 mutants cultured in higher concentration of precursors were larger than that cultured in lower concentration of precursors (Figure 3). Consequently, it seems that LuxR01 and LuxR02 exert a greater role in spoilage potential than other LuxR-type proteins. Remarkably, double mutant SB7307 showed a more significant reduction in both TMA and putrescine production (reduction to around 40%) than SB7301 and SB7302 mutants (Supplementary Figure S5).

### Biofilm Formation Ability of Deletion Strains

**FIGURE 4:**
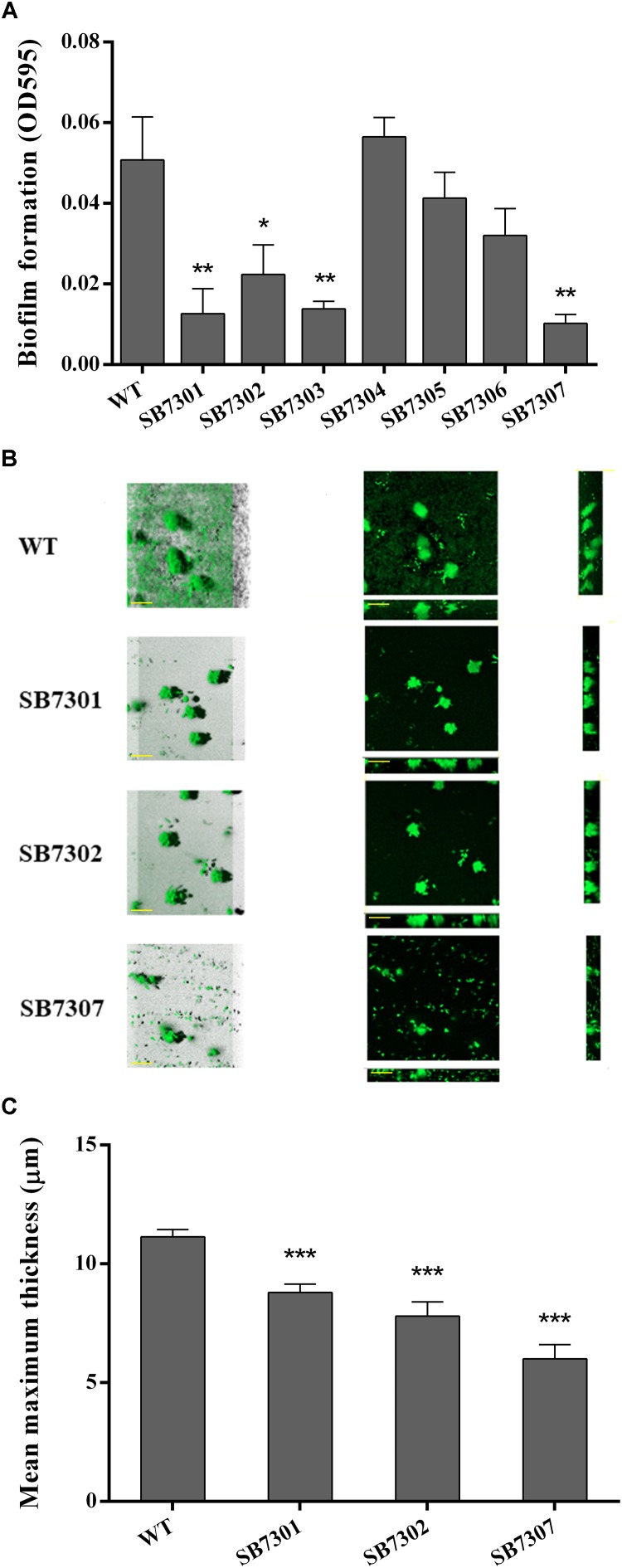
Biofilm formation activity of gene deletion mutants. **(A)** Biofilm formation of wild-type and each deletion mutant (SB7301, SB7302, SB7303, SB7304, SB7305, and SB7306) strains was quantified by microtiter plate assay. **(B)** Biofilm formation of wild-type, SB7301 and SB7302 mutant strains was analyzed by CLSM imaging in blend mode view (left panel) and section mode view (right panel). The scale bars represent 50 μm. **(C)** The mean maximum thickness of biofilms obtained from CLSM imaging. Data was presented as the mean ± standard deviation (*n* = 3, ^∗^*p* < 0.05, ^∗∗^*p* < 0.01, ^∗∗∗^*p* < 0.001).

It has been well studied that QS system is critical in regulating biofilm formation. Consequently, we investigated the regulatory role of each *luxR*-type gene in biofilm formation through microtiter plate assay. The results showed that SB7301, SB7302, and SB7303 were significantly deficient in biofilm formation, the absence of any of these genes reduced biofilm to less than half (Figure 4A). Given the excellent performance of LuxR01 and LuxR02 in PP detection and spoilage activity, we further analyzed the biofilm formation of SB7301 and SB7302 mutants by CLSM imaging. As shown in Figure 4B, the mean maximum thickness of biofilm was calculated by IMARIS software, and showed that SB7301 (*P* < 0.001) and SB7302 (*P* < 0.001) formed significantly thinner biofilm than wild-type strain, which was in accordance with the results of microtiter plate assay (Figure 4C). These results indicated that QS receptors LuxR01 and LuxR02 played important roles in spoilage potential as well as biofilm formation in *S. baltica* OS155.

### PP Promotes Biofilm Formation and Spoilage Activity in a LuxR-Type Protein-Dependent Manner

**FIGURE 5:**
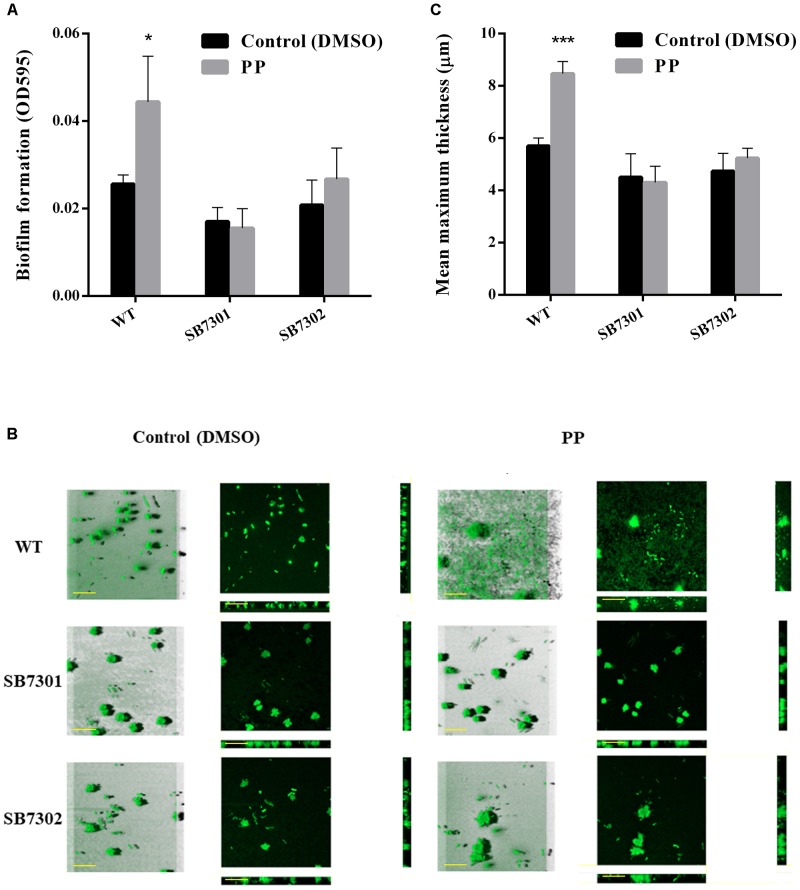
Cyclo-(L-Pro-L-Phe) promotes biofilm formation in a LuxR-type protein-dependent manner. **(A)** Biofilm formation of wild-type strain, SB7301 and SB7302 mutants with DMSO (control) and PP treatment was quantified by microtiter plate assay. **(B)** Biofilm formation of wild-type strain, SB7301 and SB7302 mutants with DMSO (control) and PP treatment was analyzed by CLSM imaging in blend mode view (left panel) and section mode view (right panel). The scale bars represent 50 μm. **(C)** The mean maximum thickness of biofilm formed in wild-type strain, SB7301 and SB7302 mutants with DMSO (control) and PP treatment was calculated by IMARIS software. Data was presented as the mean ± standard deviation (*n* = 3, ^∗^*p* < 0.05, ^∗∗∗^*p* < 0.001).

**FIGURE 6:**
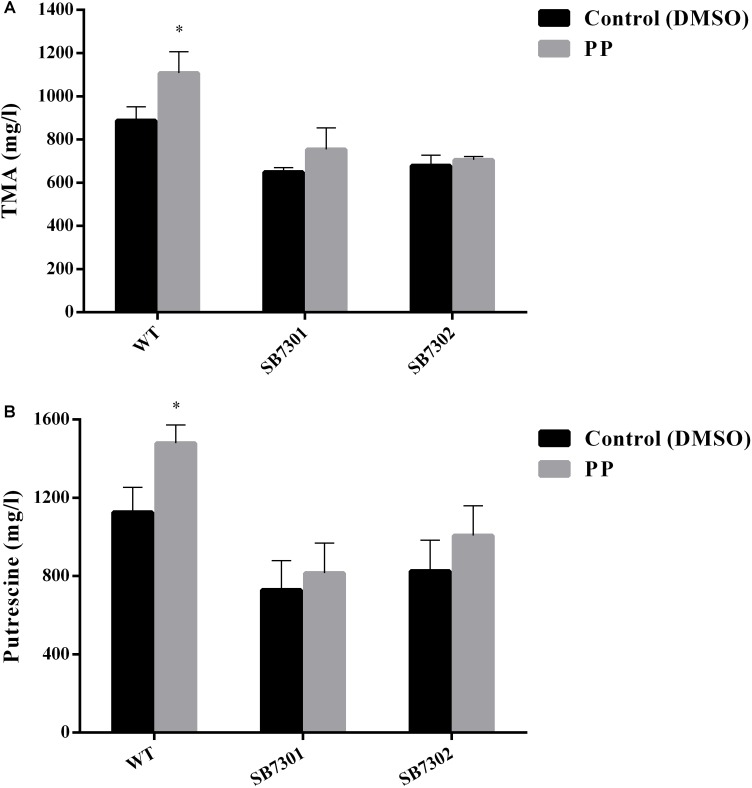
Cyclo-(L-Pro-L-Phe) promotes spoilage activity in a LuxR-type protein-dependent manner. The TMA **(A)** and putrescine **(B)** production of wild-type strain, SB7301 and SB7302 mutants with DMSO (control) and PP treatment were measured. Data was presented as the mean ± standard deviation (*n* = 3, ^∗^*p* < 0.05, ^∗∗^*p* < 0.01).

According to our previous results, PP could be recognized by cell density-dependent proteins LuxR01 and LuxR02, which were found to be responsible for spoilage activity and biofilm formation. Then we attempt to verify whether these LuxR-type proteins exert their function via interaction with PP molecule. To that end, we added exogenous PP to the culture system of wild-type strain, SB7301 and SB7302 mutants and measured their spoilage and biofilm formation activity. Both microtiter plate assay and CLSM imaging showed that PP significantly facilitated the biofilm formation in wild-type strain but not SB7301 and SB7302 mutants (Figure 5). Besides, PP partially enhanced the spoilage activity of *S. baltica* OS155, which could be attenuated by the deletion of *luxR01* or *luxR02* gene (Figure 6). These data suggested that LuxR01 and LuxR02 interacted with PP synergistically to mediate the QS-induced phenotypes such as biofilm formation and spoilage potential in different degrees.

### Transcription Profiling in SB7301 and SB7302 Mutants

**FIGURE 7:**
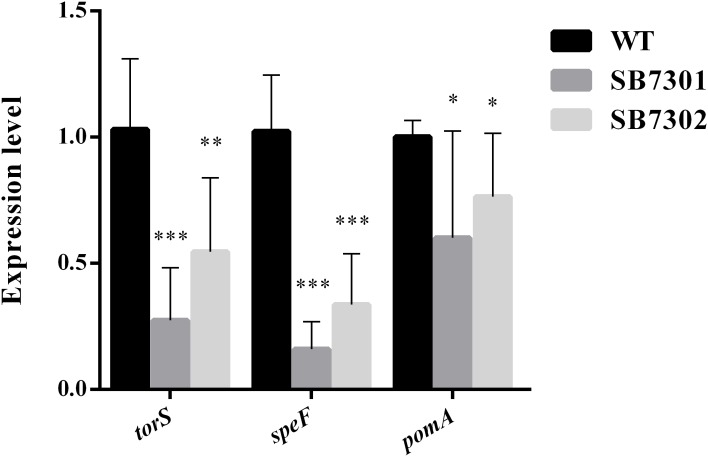
Transcription verification in gene deletion mutants. The expression level of *torS, speF*, and *pomA* genes in wild-type strain, SB7301 and SB7302 mutants were quantified by qRT-PCR. Data was presented as the mean ± standard deviation (*n* = 3, ^∗^*p* < 0.05, ^∗∗^*p* < 0.01, and ^∗∗∗^*p* < 0.001).

We have demonstrated that PP molecule enhanced spoilage activity partially and biofilm formation in *S. baltica* OS155 through the direct interaction with LuxR01 or LuxR02 proteins. To further investigate the detailed mechanism of DKP-LuxR type QS system in regulating spoilage activity and biofilm formation, high-throughput transcriptome analysis was used to identify the downstream genes regulated by LuxR01 and LuxR02 proteins. We first chose SB7301 mutant unbiasedly to be subject to transcriptome analysis. A total of 335 genes exhibited significant changes in expression level compared with wild-type strain were found, including 197 upregulated genes and 138 downregulated genes (Supplementary Data Set S1). These differentially expressed genes were further grouped according to the pathway they involved in based on the KEGG (Kyoto encyclopedia of genes and genomes) database. Results showed that these genes mainly participated in amino acid metabolism, lipid metabolism, carbohydrate metabolism, energy metabolism, signal transduction, cell motility and membrane transport (Supplementary Figure S6), which were related to spoilage potential to varying degrees. Among them, *torS* was responsible for TMA production (Jourlin et al., 1996), *speF* was responsible for putrescine production (Kashiwagi et al., 1991), and *pomA* was involved in biofilm formation (McCarter, 2001; Thormann et al., 2004). According to KEGG database, *torS* and *pomA* belong to two-component system, and *speF* belong to arginine and proline metabolism system (Supplementary Figure S7). All of these genes were significantly downregulated inSB7301 and SB7302 mutants compared with wild-type strain and was verified by qRT-PCR (Figure 7). These results revealed that DKP-LuxR type QS system is responsible for spoilage activity and biofilm formation in *S. baltica* OS155 via the transcriptional regulation of *torS, speF*, and *pomA* genes.

## Discussion

LuxR-type receptors as cytoplasmic transcription factors together with AHLs synthesized by cognate LuxI-type synthases are commonly used in Gram-negative bacteria and have been intensively studied (Kievit and Iglewski, 2000; Reading and Sperandio, 2006; Papenfort and Bassler, 2016). In this study, three DKPs (PP, PL, and LL) were detected predominant in cell-free culture fluids of *S. baltica* OS155 as well as spoiled large yellow croaker, moreover, we failed to detect any AHLs in pure culture of *S. baltica* OS155, which was largely consistent with previous studies (Gu et al., 2013; Zhu et al., 2015, 2016). According to a recent survey presented by Hudaiberdiev et al. (2015), an unexpectedly high number of annotated LuxR proteins in NCBI databases were found to be orphans lacking *luxI* genes in their vicinity on the chromosome, and thus it was speculated that new signaling molecules rather than AHLs may interact with orphan LuxR proteins (Hudaiberdiev et al., 2015). On the basis of these supporting data described above, our work took the interaction between DKPs and orphan LuxR proteins as a focal point to explore QS system and concomitantly the underlying mechanism of how QS regulates the spoilage potential and biofilm formation in *S. baltica* OS155.

Based on the cell density-dependent principle, we demonstrated that the orphan *luxR*-type genes (*luxR01, luxR02, luxR03, luxR05*, and *luxR06*) were upregulated in different degrees by postexponential supernatant extract of *S. baltica* OS155 with statistical significance, indicating their involvement in QS system preliminarily. We found that PP could interact with orphan LuxR01 and LuxR02 proteins directly, and contrastively other LuxR-type proteins employed in our study were failed to bind any hypothetical autoinducers employed. However, their cell density-dependent expression suggests the existence of other autoinducers in *S. baltica* OS155. Moreover, results showed that only SB7301 and SB7302 mutants had defects in all the tested spoilage indexes simultaneously, and SB7307 mutant had a more significant reduction in spoilage potential. Since the reduction of TMA and putrescine production of SB7301 and SB7302 mutants was not so large when using 10 mM and 0.5% precursors, a higher concentration of substrates was used for further analysis. The larger reduction of TMA and putrescine production in SB7301 and SB7302 mutants cultured in higher concentration of precursors indicated that limited the reaction as well as the generation of product is substrates rather than the enzymes with low concentration of substrates. Combined with our *in vitro* binding assay results, we propose that LuxR01 and LuxR02 proteins were DKP-dependent QS receptors as well as key biofilm regulators, and putrescine production were also dual-regulated by LuxR01 and LuxR02. However, the specific ratio of LuxR01 and LuxR02 in regulating these phenotypes and the function of other LuxRs tested here need further study. In addition, according to the conserved domain of LuxRs (Supplementary Figure S1), LuxR02 has a response regulator domain which may receive the signal from the sensor partner in bacterial two-component systems like the canonical QS systems in *Vibrio harveyi* and *V. cholera* (Papenfort and Bassler, 2016), indicating that LuxR02 may participate in a two-component QS system with novel extracellular signal compounds and regulate corresponding gene expressions in *S. baltica* OS155. Further work should be done to reveal the specific roles of other orphan LuxR-type proteins and whether LuxR01 interact with LuxR02 to regulate these phenotypes. The proposed model was further confirmed via exogenous addition of synthetic PP to wild-type, SB7301 and SB7302 mutant strains. Results exhibited different degrees of increase in TMA, putrescine production and biofilm formation in wild-type strain rather than the two mutants, indicating that the interaction between PP and LuxR01 as well as LuxR02 receptors was involved in controlling spoilage potential and biofilm formation in *S. baltica* OS155. On the contrary, two previous reports demonstrated that exposure to exogenous PL but not PP promoted spoilage-related metabolites and biofilm formation in *S. baltica* (Zhu et al., 2015, 2016). This contradiction may result from the different strains we used. Up to now, there have been 10 genomes of *S. baltica* species in NCBI database ^1^, the difference between these strains is listed in Supplementary Data Set S2. The strain used in our study is *S. baltica* OS155 which shows 98% identity and 87% query cover in genome with the representative strain *S. baltica* OS678. On the other hand, the concentration of DKPs they used (100 μM) was an order of magnitude larger than that of DKPs used here (10 μM), which was chosen our experiments to better mimic *in vivo* environment based on the quantification of PP in *S. baltica* OS155 (Table 1). It is possible that different DKPs have different optimum concentrations, and a higher concentration may have some side effects, e.g., the optimal concentration range for their activity will be revealed by further study. Although different strains may employ different mechanisms, all of these results indicate a role of DKPs in adjusting physiological behaviors such as spoilage potential and biofilm formation.

The reduction of trimethylamine oxide (TMAO) into TMA regulated by the *torCAD* operon is only induced in the presence of TMAO (Jourlin et al., 1996). The *tor* operon is regulated by its upstream genes, *torS, torT*, and *torR*, and disruption of each one can influence *tor* operon induction. The *torS* and *torR* genes encode a two-component regulatory system in which TorS is an unorthodox sensor and TorR is a response regulator (Jourlin et al., 1996). TorT interacts with TMAO upon its appearance to form the TorT-TMAO complex which further interacts with the TorS sensor, then TorS undergoes a conformational change to act as a kinase (Baraquet et al., 2006), which transphosphorylates TorR via a four-step phosphorelay (Jourlin et al., 1997). Once phosphorylated, TorR activates the expression of *tor* operon (Simon et al., 1995). In this study, we demonstrated that *torS* as a signal-transduction cascade mediator was transcriptionally regulated by LuxR-type receptors LuxR01 and LuxR02, which provides evidence connecting QS with spoilage-related gene. It is noteworthy that *torS* rather than *torA* was regulated by QS system, the indirect regulation of TorA amplified the QS signal and ensured the precise expression of TorA only at the presence of TMAO, avoiding the waste of energy when TMAO is absent.

The function of *speF* was well studied in *E. coli*, the *speF*-*potE* operon encoding an inducible ornithine decarboxylase and an ornithine/putrescine antiporter is induced at acidic pH, the ornithine decarboxylase activity was observed in the cells cultured at pH 5.2, but not at pH 7.0 (Kashiwagi et al., 1991). Since the internal pH was close to 7.0 in unstressed cells (Wilks and Slonczewski, 2007), *E. coli* utilized multiple mechanisms to ensure that SpeF remained inactive to reduce the consumption of amino acids around neutral pH, while constitutive ornithine decarboxylase was present (Kanjee et al., 2011). Our study showed that *speF* gene was significantly downregulated in SB7301 and SB7302 mutants as compared to wild-type strain. The regulation of the inducible ornithine decarboxylase but not the constitutive ornithine decarboxylase by LuxR-type proteins allowed conclusion to be drawn that QS controlled the putrescine production of *S. baltica* via consumption of amino acids in a well-organized and energy-saving way. However, it remains unknown whether the QS-dependent regulation of SpeF expression is in parallel with the acid stress-dependent regulation.

Flagella are supposed to play a key role in the initial steps of adhesion of bacteria to surfaces and biofilm formation (Kogure and Ikemoto, 1998; McCarter, 2001). Flagella rotation are powered by flagellar motor which is responsible for relaying the transmembrane proton or sodium ion gradient into energy (Hirota and Imae, 1983; Blair, 2003). Two cytoplasmic membrane proteins, MotA and MotB or PomA and PomB, form a complex at a molar ratio of 2: 1 as the stator of motor to conduct ions and generate the torque for rotation (Sato and Homma, 2000; McCarter, 2001; Berg, 2003; Kojima and Blair, 2004). In this study, the *pomA* gene was significantly downregulated in SB7301 and SB7302 mutants. PomA is homologous to MotA in *E. coli* and *Salmonella*, together with PomB to form the stator of the rotary flagellar machine. It was reported the interruption of the *pomA* but not *motA* gene yielded a non-swimming mutant and affected progression of biofilm development into pronounced three-dimensional architecture in *S. oneidensis* MR-1, and thus it was suggested that rotation of the polar flagellum was sodium dependent in analogy to *V. parahaemolyticus* where *pomA/B* potentially used a sodium gradient as energy source (McCarter, 2001; Thormann et al., 2004). In combination with exhibited biofilm phenotypes in SB7301 and SB7302 mutants, these results led to a model that LuxR-type proteins affected biofilm formation via regulating the stator component of flagellar motor PomA potentially using a sodium gradient. However, the specific relationship between biofilm formation and spoilage potential still remains unknown.

**FIGURE 8:**
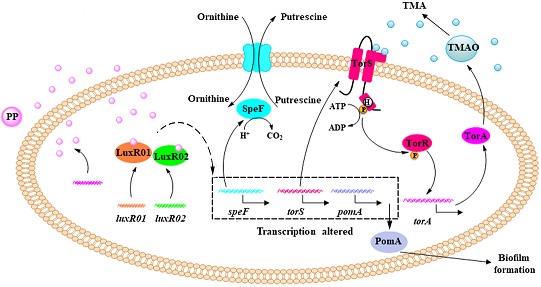
Scheme representing the QS system in regulating spoilage potential and biofilm formation in *S. baltica* OS155. At high cell density, QS signal PP reaches a threshold level and is detected by specific receptor LuxR01 and/or LuxR02 protein, which acts as transcription regulator to alter the expression of *torS, speF*, and *pomA*. Among them, TorS functions as a signal-transduction cascade mediator and transphosphorylates TorR upon the presence of TMAO, which finally activate *torA* expression to reduce TMAO into TMA. *speF* gene is responsible for the production of putrescine from ornithine. *pomA* gene promotes the biofilm formation by regulating swimming motility.

Overall, we propose a model for the mechanism of spoilage activity regulation in *S. baltica* OS155 (Figure 8). During the storage of large yellow croaker, the SSO *S. baltica* OS155 produces autoinducer molecule PP that are recognized by QS receptors LuxR01 and LuxR02, resulting in the modulation of TMA, putrescine production and biofilm formation via transcriptional regulation of *torS, speF* and *pomA*. This study provided evidence ascribing DKPs to QS autoinducers which can interact directly with orphan LuxR-type proteins, and improved our understanding of the mechanisms involved in seafood spoilage, offering new insights into improving seafood preservation based on the QS-dependent spoilage of SSO.

## Author Contributions

YW and LF designed the research. FW and CW analyzed the data. YW, FW, and CW performed the research. FW, CW, and LF wrote the manuscript. XL contributed to new reagents or analytic tools. YW developed the software necessary to perform and record experiments.

## Conflict of Interest Statement

The authors declare that the research was conducted in the absence of any commercial or financial relationships that could be construed as a potential conflict of interest.

## Footnotes

1 www.ncbi.nlm.nih.gov/genome/?term=shewanella+baltica

## Supplementary Material

FIGURE S1 The conserved domains in provisional LuxR-type proteins of *Shewanella baltica* OS155. CitB: DNA-binding response regulator, NarL/FixJ family, contains REC and HTH domains. Response_reg: response regulator receiver domain, this domain receives the signal from the sensor partner in bacterial two-component systems. It is usually found N-terminal to a DNA binding effector domain; HTH_LUXR: helix_turn_helix, Lux Regulon.

FIGURE S2 PCR verification of gene deletion mutants of *S. baltica* OS155. Deletion mutants (SB7301, SB7302, SB7303, SB7304, SB7305, and SB7306) were screened by PCR amplification using the outside primers (5′-O and 3′-O). W, wide-type strain; D, deletion mutant.

FIGURE S3 Identification and quantification of hypothetical autoinducers in *S. baltica* OS155 and spoiled large yellow croaker. AHLs and DKPs in *S. baltica* OS155 **(A)** and spoiled large yellow croaker **(B)** were measured by UHPLC-MS/MS. C4-HSL, *N*-butanoylhomoserine lactone; C6-HSL, *N*-hexanoylhomoserine lactone; PL, cyclo-(L-Pro-L-Leu); LL, cyclo-(L-Leu-L-Leu); PP, cyclo-(L-Pro-L-Phe).

FIGURE S4 Purified recombinant LuxR-type proteins. FLAG-tagged LuxR-type proteins (LuxR01–LuxR06) were expressed in *E. coli* BL21 (DE3) and purified by FLAG M2 beads. Empty plasmid pET-15b was used as a control. An aliquot of the purified proteins were qualified by SDS-PAGE with 12% **(left)** or 15% **(right)** gels. ^∗^ M2 antibody light chain.

FIGURE S5 Spoilage potential of the double mutant. The TMA **(A)** and putrescine **(B)** production of wild-type and the double mutant SB7307 strain were measured. Data was presented as the mean ± standard deviation (*n* = 3, ^∗∗^*p* < 0.01, ^∗∗∗^*p* < 0.001).

FIGURE S6 Kyoto encyclopedia of genes and genomes (KEGG) enrichment of differentially expressed genes in SB7301 mutant. The 335 differentially expressed genes are involved in amino acid metabolism, lipid metabolism, carbohydrate metabolism, energy metabolism, signal transduction, cell motility and membrane transport.

FIGURE S7 The cluster analysis diagram of differentially expressed genes belonging to two-component system and arginine and proline metabolism system in SB7301 mutant. The differential expression profile of genes involved in two-component system and arginine and proline metabolism system was summarized and presented in colored mosaic matrix. SBAL_RS16580: *torS*, SBAL_RS21090: *speF*, SBAL_RS06905: *pomA*.

TABLE S1 Bacterial strains and plasmids used in this study.

TABLE S2 Identification of target genes involved in this study in *S. baltica* OS155.

TABLE S3 LC gradient.

TABLE S4 Target genes and primers used in this study.

DATA SET S1 Annotation results of 335 differentially expressed genes.

DATA SET S2 The list of 10 strains of *S. baltica* species.

## Abbreviations

3-oxo-C10-HSL: *N*-(3-oxodecanoyl)-homoserine lactone
3-oxo-C12-HSL: *N*-(3-oxododecanoyl)-homoserine lactone
3-oxo-C14-HSL: *N*-(3-oxotetradecanoyl)-homoserine lactone
3-oxo-C8-HSL: *N*-(3-oxooctanoyl)-homoserine lactone
AHLs: *N*-acyl -homoserine lactones
BAs: biogenic amines
C10-HSL: *N*-decanoylhomoserine lactone
C12-HSL: *N*-dodecanoylhomoserine lactone
C14-HSL: *N*-tetradecanoylhomoserine lactone
C4-HSL: *N*-butanoylhomoserine lactone
C6-HSL: *N*-hexanoylhomoserine lactone
C8-HSL: *N*-octanoylhomoserine lactone
CLSM: confocal laser scanning microscopy
DAP, 2: 6-diaminopimelic acid
DKPs: diketopiperazines
Gm: gentamycin
IPTG: isopropyl-β-D-thiogalactoside
LL: cyclo-(L-Leu-L-Leu)
PL: cyclo-(L-Pro-L-Leu)
PP: cyclo-(L-Pro-L-Phe)
QS: quorum sensing
SSOs: specific spoilage organisms
TMA: trimethylamine
TMAO: trimethylamine oxide
