# Supplementary figures and images for: Positive Regulation of Spoilage Potential and Biofilm Formation in Shewanella baltica OS155 via Quorum Sensing System Composed of DKP and Orphan LuxRs
Source: Front Microbiol. 2019 Feb 5;10:135. doi: 10.3389/fmicb.2019.00135 (PMC6370745; doi:10.3389/fmicb.2019.00135)

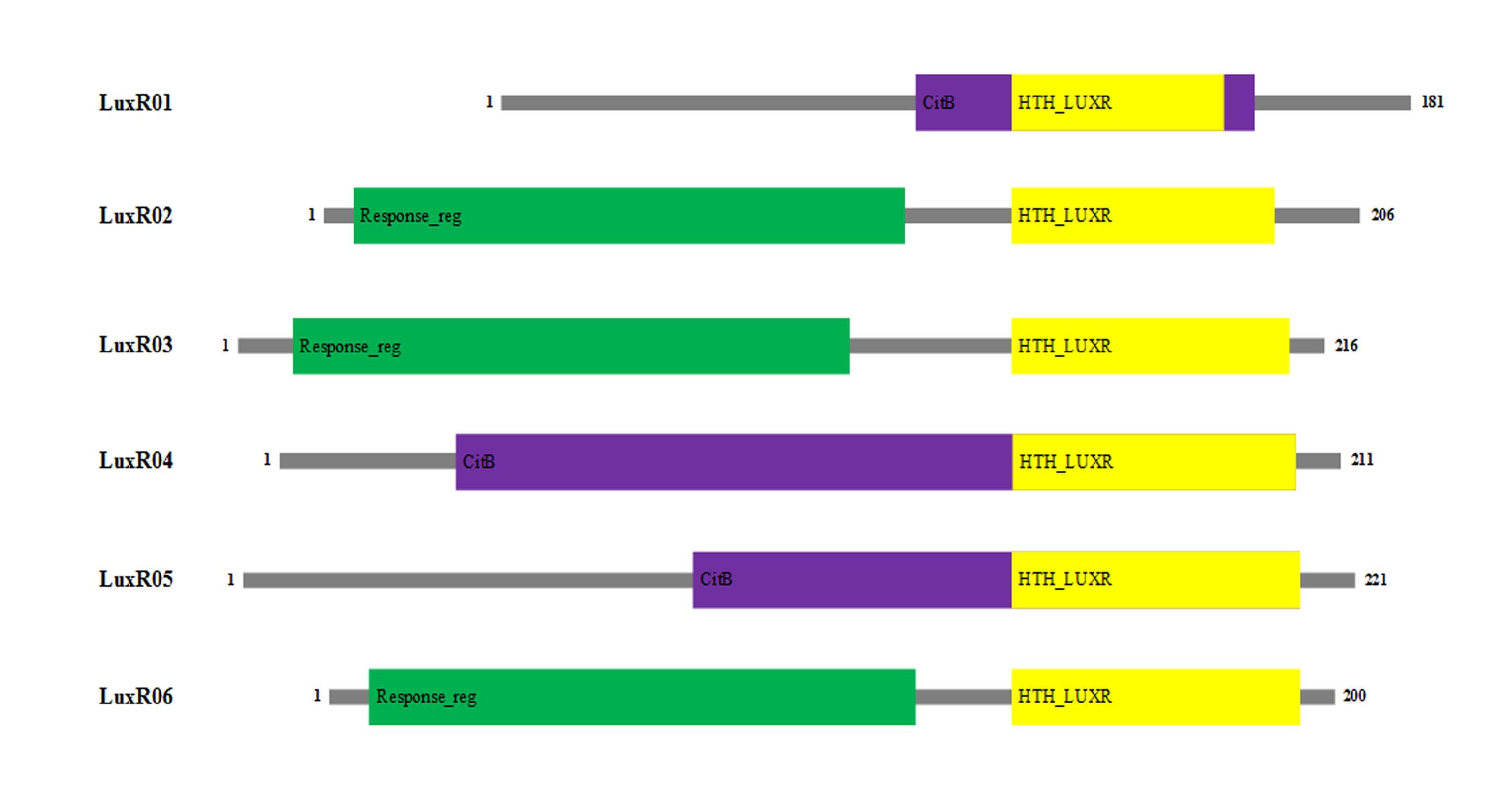

Supplement: FIGURE S1 — The conserved domains in provisional LuxR-type proteins of Shewanella baltica OS155. CitB: DNA-binding response regulator, NarL/FixJ family, contains REC and HTH domains. Response_reg: response regulator receiver domain, this domain receives the signal from the sensor partner in bacterial two-component systems. It is usually found N-terminal to a DNA binding effector domain; HTH_LUXR: helix_turn_helix, Lux Regulon. [file Image_1.TIF]

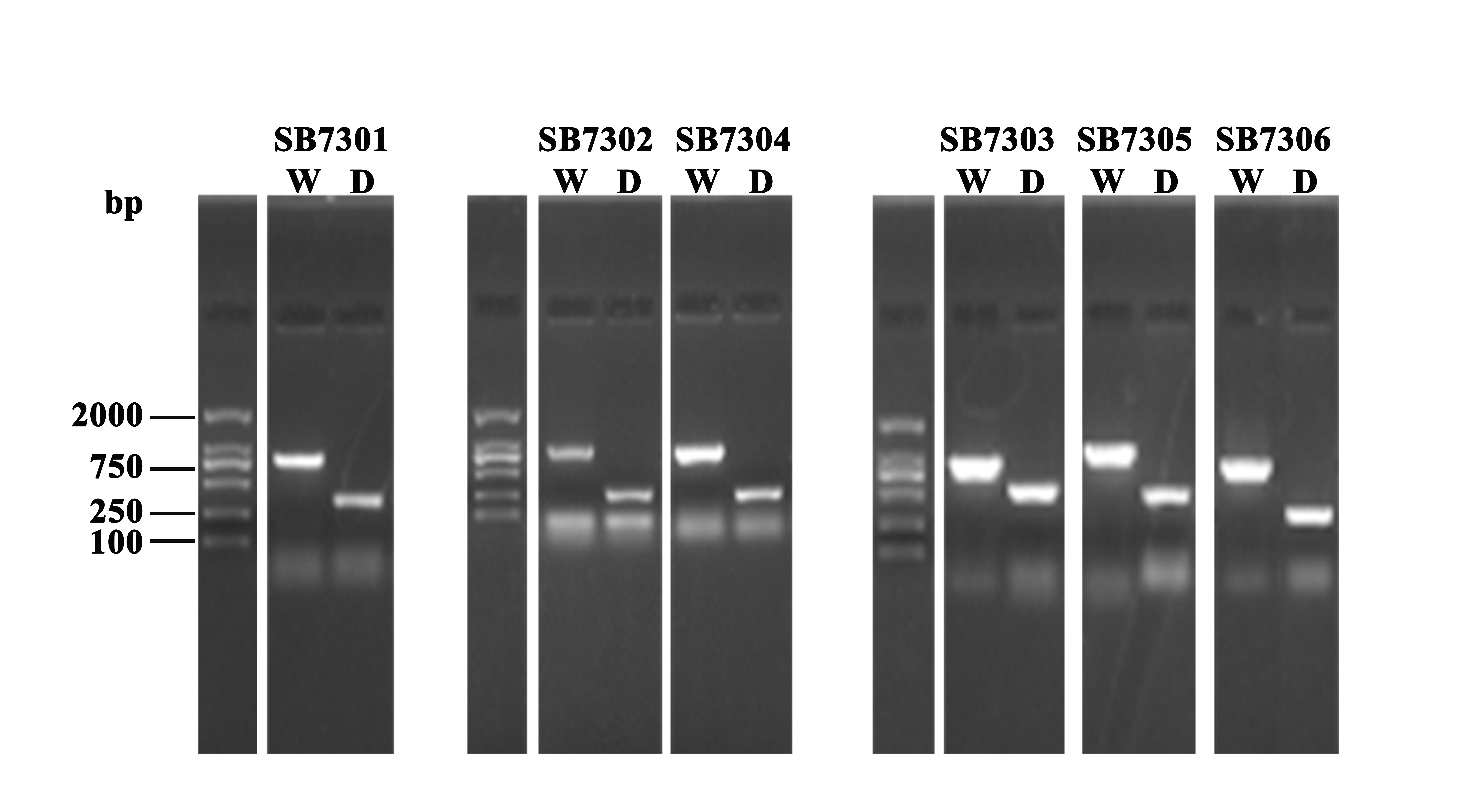

Supplement: FIGURE S2 — PCR verification of gene deletion mutants of S. baltica OS155. Deletion mutants (SB7301, SB7302, SB7303, SB7304, SB7305, and SB7306) were screened by PCR amplification using the outside primers (5′-O and 3′-O). W, wide-type strain; D, deletion mutant. [file Image_2.TIF]

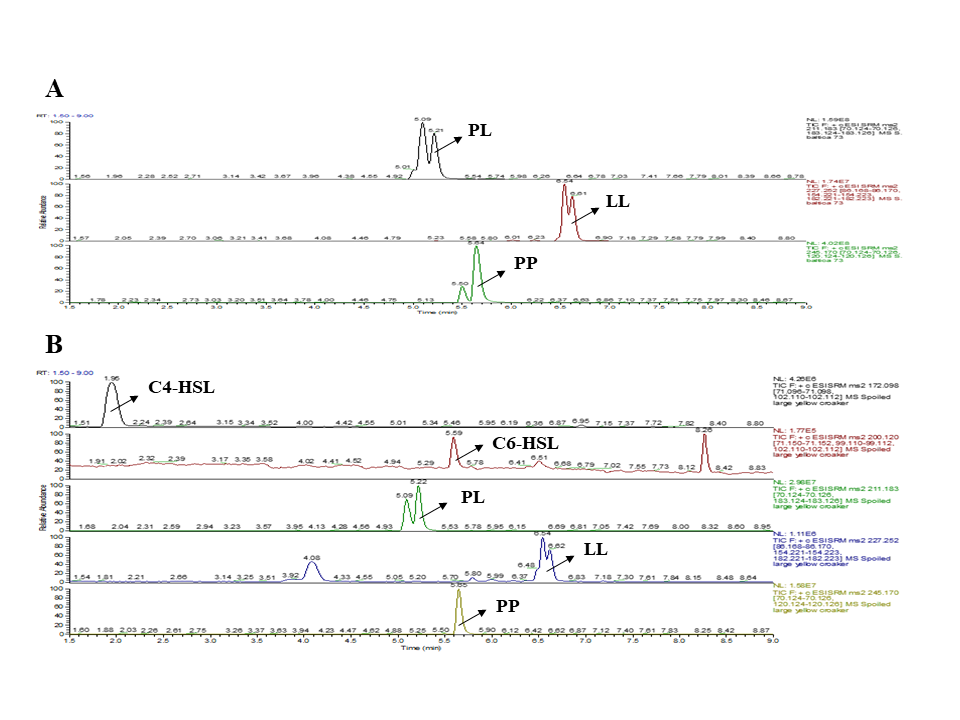

Supplement: FIGURE S3 — Identification and quantification of hypothetical autoinducers in S. baltica OS155 and spoiled large yellow croaker. AHLs and DKPs in S. baltica OS155 (A) and spoiled large yellow croaker (B) were measured by UHPLC-MS/MS. C4-HSL, N-butanoylhomoserine lactone; C6-HSL, N-hexanoylhomoserine lactone; PL, cyclo-(L-Pro-L-Leu); LL, cyclo-(L-Leu-L-Leu); PP, cyclo-(L-Pro-L-Phe). [file Image_3.TIF]

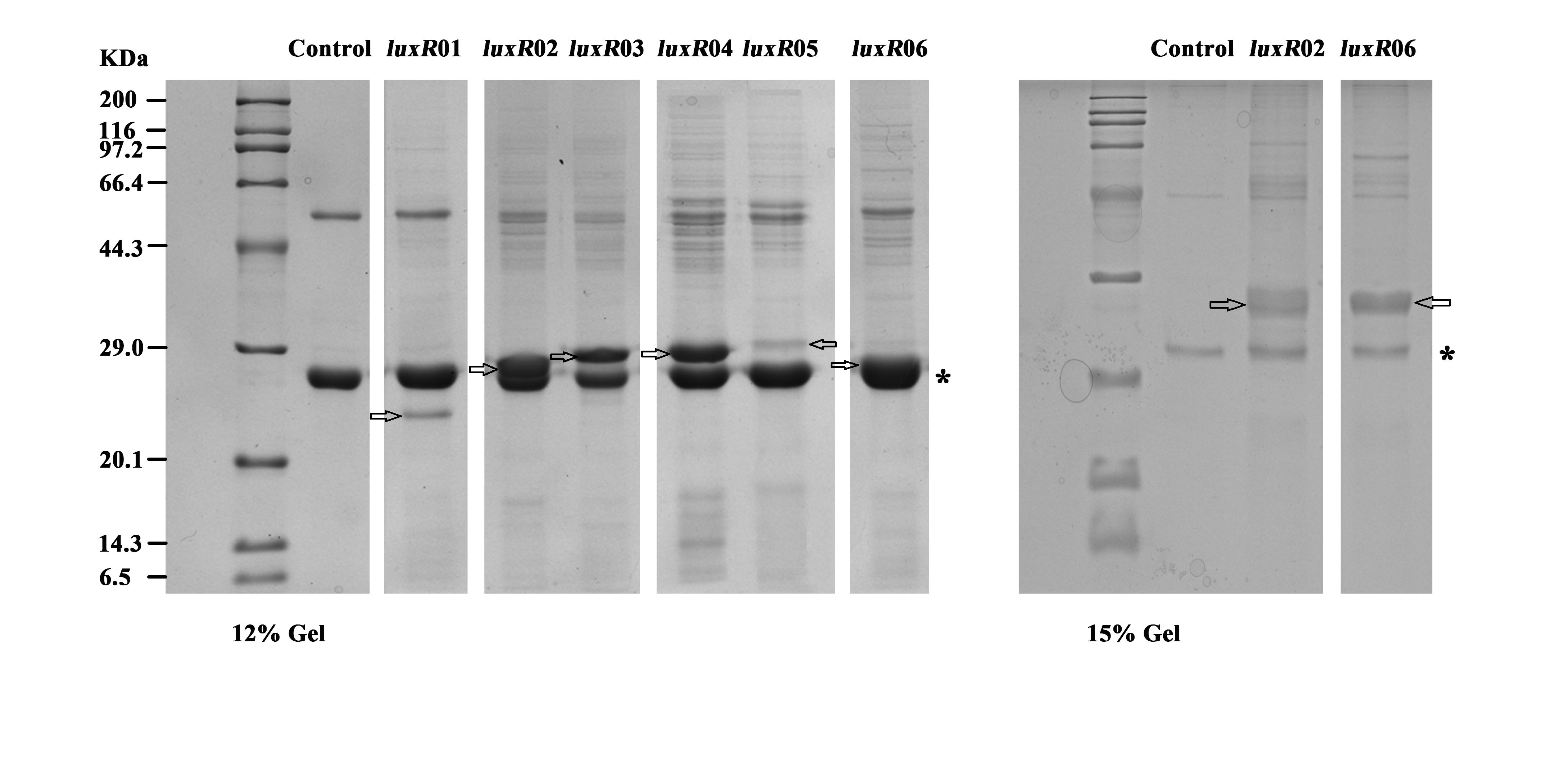

Supplement: FIGURE S4 — Purified recombinant LuxR-type proteins. FLAG-tagged LuxR-type proteins (LuxR01–LuxR06) were expressed in E. coli BL21 (DE3) and purified by FLAG M2 beads. Empty plasmid pET-15b was used as a control. An aliquot of the purified proteins were qualified by SDS-PAGE with 12% (left) or 15% (right) gels. ∗ M2 antibody light chain. [file Image_4.TIF]

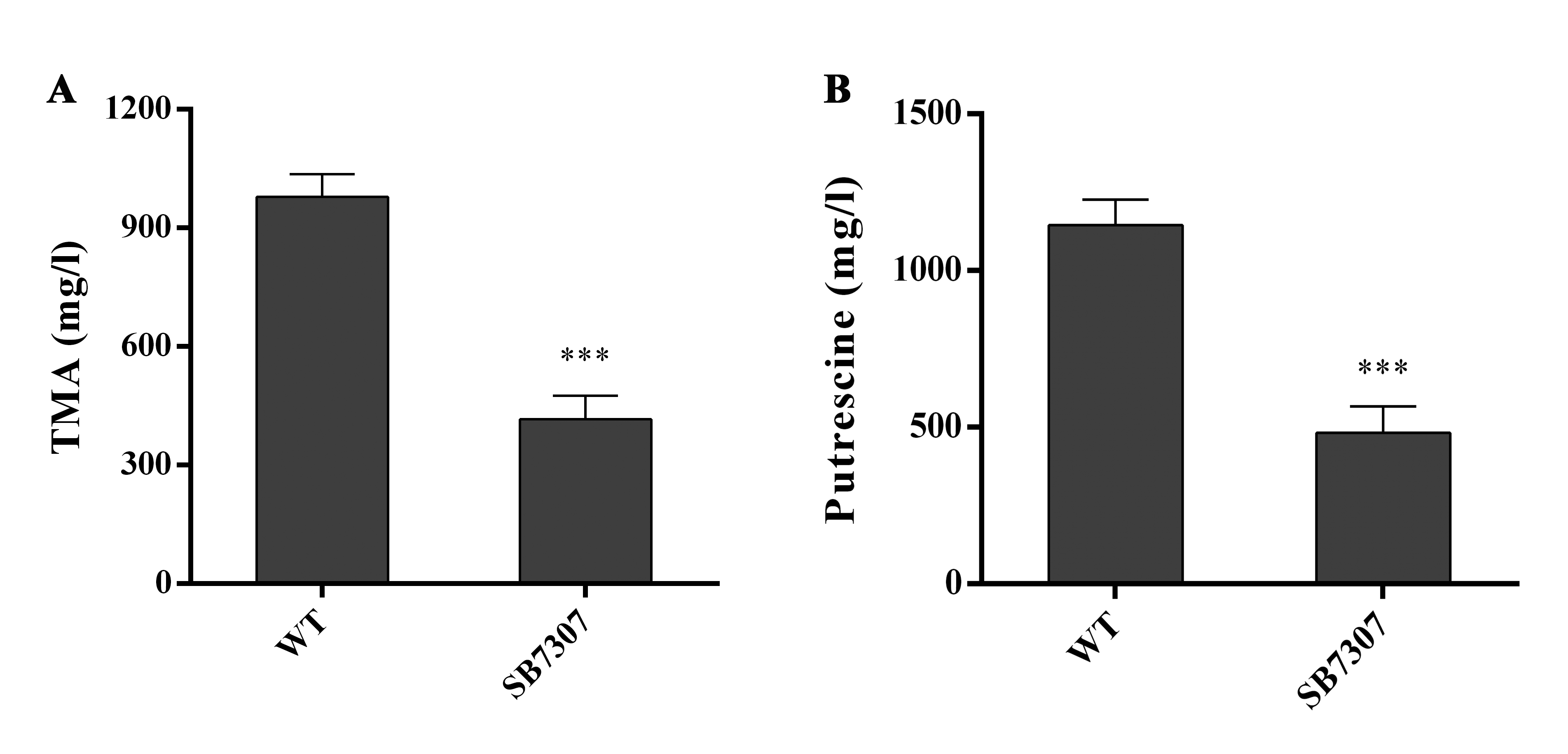

Supplement: FIGURE S5 — Spoilage potential of the double mutant. The TMA (A) and putrescine (B) production of wild-type and the double mutant SB7307 strain were measured. Data was presented as the mean ± standard deviation (n = 3, ∗∗p < 0.01, ∗∗∗p < 0.001). [file Image_5.TIF]

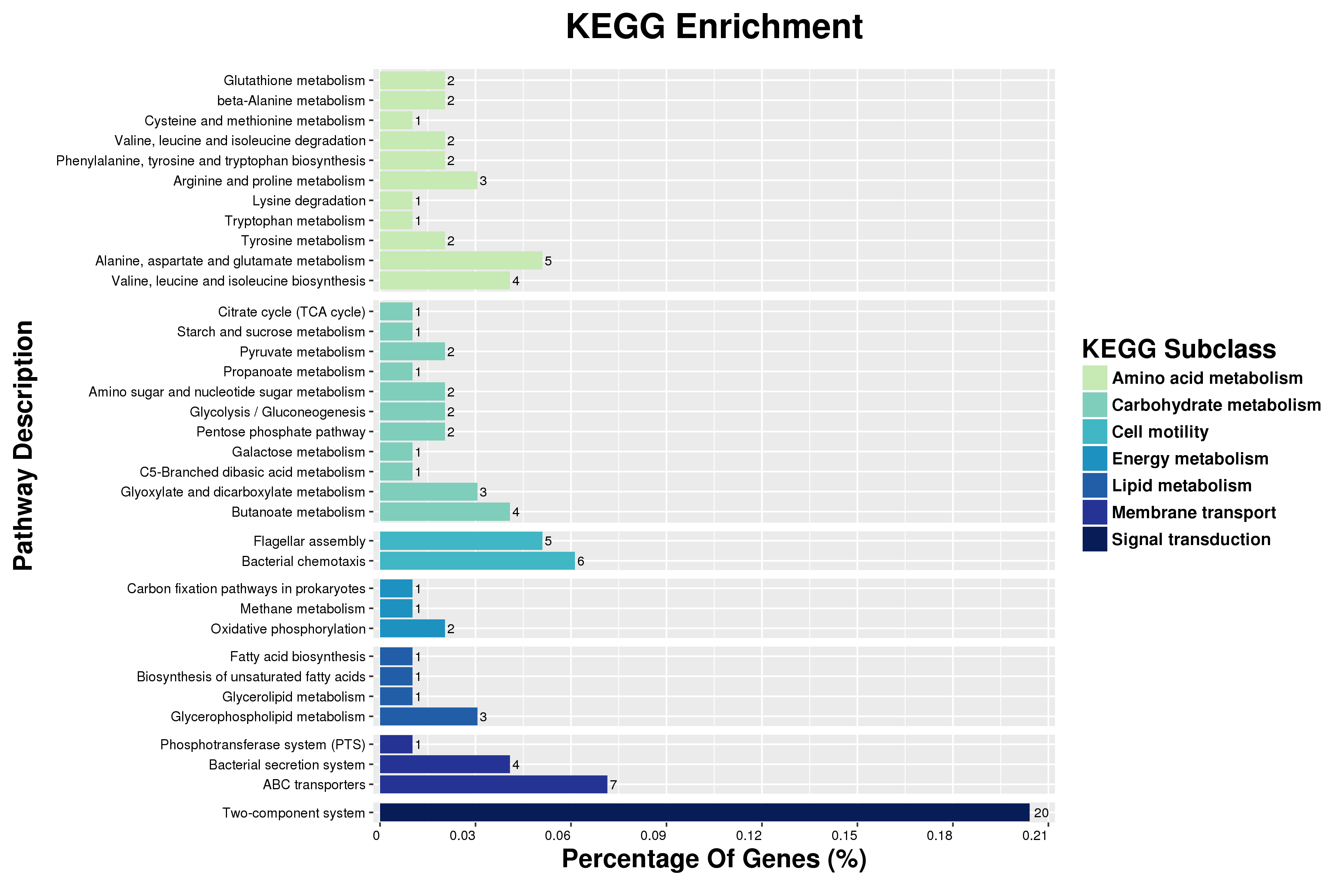

Supplement: FIGURE S6 — Kyoto encyclopedia of genes and genomes (KEGG) enrichment of differentially expressed genes in SB7301 mutant. The 335 differentially expressed genes are involved in amino acid metabolism, lipid metabolism, carbohydrate metabolism, energy metabolism, signal transduction, cell motility and membrane transport. [file Image_6.TIFF]

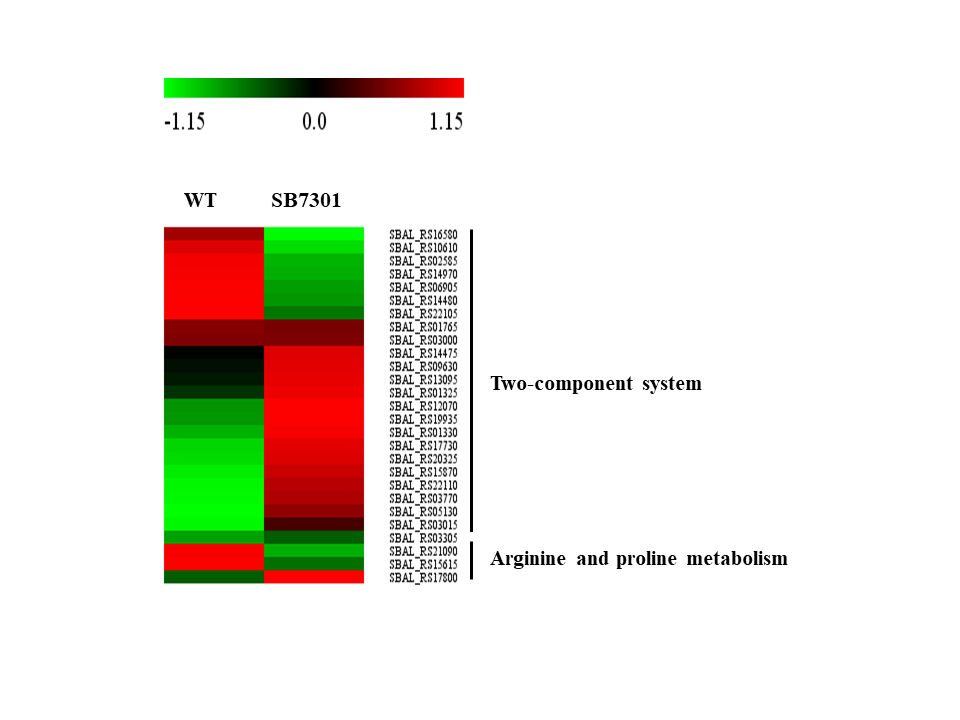

Supplement: FIGURE S7 — The cluster analysis diagram of differentially expressed genes belonging to two-component system and arginine and proline metabolism system in SB7301 mutant. The differential expression profile of genes involved in two-component system and arginine and proline metabolism system was summarized and presented in colored mosaic matrix. SBAL_RS16580: torS, SBAL_RS21090: speF, SBAL_RS06905: pomA. [file Image_7.TIF]
