# Supplementary material for: Positive Regulation of Spoilage Potential and Biofilm Formation in Shewanella baltica OS155 via Quorum Sensing System Composed of DKP and Orphan LuxRs
Source: Front Microbiol. 2019 Feb 5;10:135. doi: 10.3389/fmicb.2019.00135 (PMC6370745; doi:10.3389/fmicb.2019.00135)
Supplement: TABLE S1 — Bacterial strains and plasmids used in this study. [file Table_1.docx]

| **Table S1** Bacterial strains and plasmids used in this study | | |
| --- | --- | --- |
| Strains and plasmids | Description | Reference or source |
| *E. coli* strains |  |  |
| *E. coli* DB3.1λ | Host for pHGM01 | Originally from addgene |
| *E. coli* WM3064 | Host for *pir*-dependent plasmids and donor strain for conjugation; RP4 (*tra*) in chromosome; Δ*dapA* | Originally from BioVector NTCC |
| *E. coli* Stbl3 | Host for pET-15b Cloning host | Originally from Invitrogen |
| *E. coli* BL21 (DE3) | Expression host | Originally from BioVector NTCC |
| *S. baltica* strains |  |  |
| *S. baltica* OS155 | Wide-type | Lab stock |
| SB7301 | *S. baltica* OS155 Δ*luxR01* | This study |
| SB7302 | *S. baltica* OS155 Δ*luxR02* | This study |
| SB7303 | *S. baltica* OS155 Δ*luxR03* | This study |
| SB7304 | *S. baltica* OS155 Δ*luxR04* | This study |
| SB7305 | *S. baltica* OS155 Δ*luxR05* | This study |
| SB7306 | *S. baltica* OS155 Δ*luxR06* | This study |
| SB7307 | *S. baltica* OS155 Δ*luxR01* and Δ*luxR02* | This study |
| Plasmids |  |  |
| pHGM01 | Apr, Gmr, Cmr; mob+ att-based suicide vector | (28) |
| pET-15b | lacI, Apr, expression vector, pBR322 ori | Originally from Novagen |

| **Table S2** Identification of target genes involved in this study in *S. baltica* OS155 | | | |
| --- | --- | --- | --- |
| Target gene | Gene ID | Description | Identity with representative *S. baltica* OS678 |
|  |  |  | *S. Baltica* OS155 |
| *luxR01* | 11774396 | LuxR family transcriptional regulator | 98%  96%  97%  99%  93%  95%  99%  98%  99% |
| *luxR02* | 11773870 | DNA-binding response regulator |  |
| *luxR03* | 11774650 | DNA-binding response regulator |  |
| *luxR04* | 11772048 | Helix-turn-helix transcriptional regulator |  |
| *luxR05* | 11771179 | Helix-turn-helix transcriptional regulator |  |
| *luxR06* | 11770801 | DNA-binding response regulator |  |
| *speF* | 11774161 | Ornithine decarboxylase SpeF |  |
| *torS* | 11773397 | TMAO reductase system sensor histidine kinase TorS |  |
| *pomA* | 11771640 | Flagellar motor protein PomA |  |

| **Table S3** LC gradient | | | |
| --- | --- | --- | --- |
| Time (min) | Flow rate  (ml/min) | A% | B% |
| 0 | 0.3 | 80 | 20 |
| 0 | 0.3 | 80 | 20 |
| 1.5 | 0.3 | 80 | 20 |
| 6.0 | 0.3 | 5 | 95 |
| 7.0 | 0.3 | 5 | 95 |
| 7.3 | 0.3 | 80 | 20 |
| 9.0 | 0.3 | 80 | 20 |

| **Table S4** Target genes and primers used in this study | | |
| --- | --- | --- |
| Target genes | Purpose | Primer / Sequence |
| *luxR01* | qRT-PCR | Forward / AACTTGATGCCGCTTCATTC |
|  |  | Reverse / CAACAGGCATCGACCATTTA |
|  | Mutant construction | 5’ – O / GGGGACAAGTTTGTACAAAAAAGCAGGCTAAGAGTATGTGGGCAATCGGC |
|  |  | 5’ – I / GGTCCGGGTTCGCTATCTATGGCCAACAGACCAATAATGACAAG |
|  |  | 3’ – O / GGGGACCACTTTGTACAAGAAAGCTGGGTCCGCCACCACCATAAAACTC |
|  |  | 3’ – I / ATAGATAGCGAACCCGGACCGGCGGCTTAATCTGCGCT |
|  | Protein expression | Forward / GTGCCGCGCGGCAGCCATATGAAGGATCTTGTCATTATTGGTCTGTTG |
|  |  | Reverse / GCTTTGTTAGCAGCCGGATCCTTACTTGTCATCGTCGTCCTTGTAGTCGCCGCC AGCCGCCTTAGAACTTGATGC |
| *luxR02* | qRT-PCR | Forward / TCGCCGATAGCCTCTGTATT |
|  |  | Reverse / GCGATCCGGCTATAGATCAG |
|  | Mutant construction | 5’ – O / GGGGACAAGTTTGTACAAAAAAGCAGGCTACGAACTGAATCAACCTTTGGC |
|  |  | 5’ – I / GGTCCGGGTTCGCTATCTATTAAAGTGCTCCATTAGGTTCAGG |
|  |  | 3’ – O / GGGGACCACTTTGTACAAGAAAGCTGGGTCATGGTGTGGCACAGAATAGTGTC |
|  |  | 3’ – I / ATAGATAGCGAACCCGGACCACGCCTGCGCCTTGAAGAT |
|  | Protein expression | Forward / GTGCCGCGCGGCAGCCATATGAGTCTTGAGTTACCTGTGTATTTAATCG |
|  |  | Reverse / GCTTTGTTAGCAGCCGGATCCTTACTTGTCATCGTCGTCCTTGTAGTCGCCGCC GCGGTTATCTTCAAGGCGC |
| *luxR03* | qRT-PCR | Forward / CAATTGGTTCGCCAAGGTAT |
|  |  | Reverse / CGACAGGATTATTGGCGAGT |
|  | Mutant construction | 5’ – O / GGGGACAAGTTTGTACAAAAAAGCAGGCTTCGGCCACCAGCTCACAG |
|  |  | 5’ – I / GGTCCGGGTTCGCTATCTATCAATTGTTGATCTTCTACTAAGCCG |
|  |  | 3’ – O / GGGGACCACTTTGTACAAGAAAGCTGGGTAGGAAGTGTTAGCGCACTAGTGTG |
|  |  | 3’ – I / ATAGATAGCGAACCCGGACCACCCAATAGCATTCAGCAAGACC |
|  | Protein expression | Forward / GTGCCGCGCGGCAGCCATATGGATACGCAAAACAACATCATCAGTG |
|  |  | Reverse / GCTTTGTTAGCAGCCGGATCCTTACTTGTCATCGTCGTCCTTGTAGTCGCCGCC GATCAATGCCAGTTGTAAGCC |
| *luxR04* | qRT-PCR | Forward / TTAATGCAAGCAGCCACAAC |
|  |  | Reverse / GATCACCCTATGGCCTCGTA |
|  | Mutant construction | 5’ – O / GGGGACAAGTTTGTACAAAAAAGCAGGCTGGCCAGTTCTGACTTGTAGGTCA |
|  |  | 5’ – I / GGTCCGGGTTCGCTATCTATGAACATCCTGCTCACTCCCTGT |
|  |  | 3’ – O / GGGGACCACTTTGTACAAGAAAGCTGGGTATGGCAGGAATATGCTCTGCA |
|  |  | 3’ – I / ATAGATAGCGAACCCGGACCGGCGCAGACCTATTTGCACG |
|  | Protein expression | Forward / GTGCCGCGCGGCAGCCATATGTTCAAAATATTACATTGGATTGTAGTGTC |
|  |  | Reverse / GCTTTGTTAGCAGCCGGATCCTTACTTGTCATCGTCGTCCTTGTAGTCGCCGCC  AACCACCACATCGTGCAAATA |
| *luxR05* | qRT-PCR | Forward / AACAACAGGCCACGAATACC |
|  |  | Reverse / GGGATCCCTTTACTGGCTTC |
|  | Mutant construction | 5’ – O / GGGGACAAGTTTGTACAAAAAAGCAGGCTATGAACTCATCGAATGGTGGG |
|  |  | 5’ – I / GGTCCGGGTTCGCTATCTATGCGAGTAACGCTAAATGACATGGTG |
|  |  | 3’ – O / GGGGACCACTTTGTACAAGAAAGCTGGGTGGCGCATACGGCACAGATAAC |
|  |  | 3’ – I / ATAGATAGCGAACCCGGACCCGGTGAAGGCTCACATTTCC |
|  | Protein expression | Forward / GTGCCGCGCGGCAGCCATATGAGCACAATTAATGAATTAGTTTTTTTAC |
|  |  | Reverse / GCTTTGTTAGCAGCCGGATCCTTACTTGTCATCGTCGTCCTTGTAGTCGCCGCC  GCTGCACAGCTCAAAGTGGG |
| *luxR06* | qRT-PCR | Forward / TGCTTATTGGTCATGCCTTG |
|  |  | Reverse / AAAAGGCACAGCGTGAAAGT |
|  | Mutant construction | 5’ – O / GGGGACAAGTTTGTACAAAAAAGCAGGCTGGCCTGCGGATCACTTGC |
|  |  | 5’ – I / GGTCCGGGTTCGCTATCTATCATGGATTTCCTTATGGAGGCG |
|  |  | 3’ – O / GGGGACCACTTTGTACAAGAAAGCTGGGTCCCTATGGCAAGGAGCGC |
|  |  | 3’ – I / ATAGATAGCGAACCCGGACCGCTAGTGCAGCATCTCGCAAACC |
|  | Protein expression | Forward / GTGCCGCGCGGCAGCCATATGTCTACAAAATTGCCACTGTATTTAGTCG |
|  |  | Reverse / GCTTTGTTAGCAGCCGGATCCTTACTTGTCATCGTCGTCCTTGTAGTCGCCGCC AATGGGAGTGTTTAGGTTTGC |
| *torS* | qRT-PCR | Forward / CATCGACCGTGAATGACCTG |
|  |  | Reverse / CCTGCGCTAACTGCTTGAGA |
| *speF* | qRT-PCR | Forward / CATGCATCATAAAGGCGTTG |
|  |  | Reverse / TCAGTCGGTGCATAAACAGC |
| *pomA* | qRT-PCR | Forward / GCATGATTGGTACGCTGGTG |
|  |  | Reverse / GCGACCATAGCGCCGTATAA |
